# A Cloud Computing-Based Modified Symbiotic Organisms Search Algorithm (AI) for Optimal Task Scheduling

**DOI:** 10.3390/s22041674

**Published:** 2022-02-21

**Authors:** Ajoze Abdulraheem Zubair, Shukor Abd Razak, Md. Asri Ngadi, Arafat Al-Dhaqm, Wael M. S. Yafooz, Abdel-Hamid M. Emara, Aldosary Saad, Hussain Al-Aqrabi

**Affiliations:** 1 Faculty of Engineering, School of Computing, Universiti Teknologi Malaysia (UTM), Johor Bahru 81310, Malaysia; shukorar@utm.my (S.A.R.); dr.asri@utm.my (M.A.N.); mrarafat1@utm.my (A.A.-D.); 2 Department of Computer Science, College of Computer Science and Engineering, Taibah University, Medina 42353, Saudi Arabia; wyafooz@taibahu.edu.sa (W.M.S.Y.); aemara@taibahu.edu.sa (A.-H.M.E.); 3 Department of Computers and Systems Engineering, Faculty of Engineering, Al-Azhar University, Cairo 11884, Egypt; 4 Computer Science Department, Community College, King Saud University, Riyadh 11437, Saudi Arabia; saldosary@ksu.edu.sa; 5 Department of Computer Science, School of Computing and Engineering, University of Huddersfield, Queensgate, Huddersfield HD1 3DH, UK; h.al-aqrabi@hud.ac.uk

**Keywords:** cloud computing, cloud resource management, task scheduling, ecosystem, geometric mean, symbiotic organisms search algorithm, convergence speed

## Abstract

The search algorithm based on symbiotic organisms’ interactions is a relatively recent bio-inspired algorithm of the swarm intelligence field for solving numerical optimization problems. It is meant to optimize applications based on the simulation of the symbiotic relationship among the distinct species in the ecosystem. The task scheduling problem is NP complete, which makes it hard to obtain a correct solution, especially for large-scale tasks. This paper proposes a modified symbiotic organisms search-based scheduling algorithm for the efficient mapping of heterogeneous tasks to access cloud resources of different capacities. The significant contribution of this technique is the simplified representation of the algorithm’s mutualism process, which uses equity as a measure of relationship characteristics or efficiency of species in the current ecosystem to move to the next generation. These relational characteristics are achieved by replacing the original mutual vector, which uses an arithmetic mean to measure the mutual characteristics with a geometric mean that enhances the survival advantage of two distinct species. The modified symbiotic organisms search algorithm (G_SOS) aims to minimize the task execution time (makespan), cost, response time, and degree of imbalance, and improve the convergence speed for an optimal solution in an IaaS cloud. The performance of the proposed technique was evaluated using a CloudSim toolkit simulator, and the percentage of improvement of the proposed G_SOS over classical SOS and PSO-SA in terms of makespan minimization ranges between 0.61–20.08% and 1.92–25.68% over a large-scale task that spans between 100 to 1000 Million Instructions (MI). The solutions are found to be better than the existing standard (SOS) technique and PSO.

## 1. Introduction

Cloud computing is a modern computing model that offers the virtualization of computing services as a utility to Cloud service users [1,2,3,4]. It is a concept for obtaining resources from a customizable shared resource, such as a group of networks, servers, storage, utilities, and applications, instantaneously and based on request. Cloud service providers use virtualization technologies to utilize resources better by allowing multiple virtual machines (VMs) to operate on top of a single physical computer. Consumers of cloud services are automatically provisioned based on Service-Level Agreements (SLA), which are usually formed by negotiations between Cloud service providers and Cloud service users/consumers. Issues related to inefficient mapping of tasks to cloud resources often occur in a cloud environment [5,6,7,8].

Task scheduling, therefore, refers to the efficiently scheduling of computational activities and rational allocation of computing resources under some restrictions in the IaaS cloud environment. Scheduling’s job is to assign tasks to the most suitable resources in order to achieve one or more goals. Therefore, selecting an appropriate work scheduling algorithm to increase cloud computing resource efficiency, while keeping high quality of service (QoS) guarantees, is an important issue that continues to attract research attention [9,10,11].

As a result of the broad solution space and the complex existence of heterogeneous resources in cloud computing, the task scheduling problem falls into the group of NP-hard issues [12,13,14,15].

Heuristic and metaheuristic scheduling techniques have been used to address the task scheduling problem in cloud computing [2,16]. For more trivial concerns, heuristic methods provide optimal results, but, as the size of the problem rises, the solutions generated by these algorithms will be less optimal. On the other hand, metaheuristic algorithms have shown impressive effectiveness in delivering near-optimal scheduling solutions for a complex large-size problem. In recent years, an increasing number of independent scholars have investigated the quality of service provided by task scheduling techniques. Several metaheuristic strategies, such as Artificial Bee Colony (ABC) Algorithm, Particle Swarm Optimization (PSO), Ant Colony Optimization (ACO), Genetic Algorithm (GA), Symbiotic Organisms Search (SOS), Cuckoo Search (CS), and Flower Pollination Algorithm (FPA), have been developed to address various challenges, such as inefficient cost, high execution time, and SLA parameters to be fulfilled by cloud service providers as stated by cloud consumers. A hybrid multi-objective Artificial Bee Colony Algorithm is used in a cloud computing system to solve flexible work scheduling challenges [17]. The proposed technique models the challenges as a hybrid flowshop (HFS) problem into HFS with identical parallel machines and HFS with heterogeneous machines. In addition, several different types of perturbation structures are examined to improve the searching capabilities. An updated version of the adaptive perturbation structure is integrated into the proposed technique to balance the exploitation and exploration capability. 

In [18], a cloud computing task scheduling based on a hybrid particle swarm algorithm and an ant colony algorithm is presented. The integrated algorithm can maintain a specified concentration of particles in the fitness level while ensuring population diversity. Furthermore, the global best solution with high accuracy convergence may be obtained precisely by adjusting the learning factor. Similarly, an approach to adaptive load balanced task scheduling in cloud computing has been presented by [19]. The proposed technique hybridized the ACO algorithm and Genetic Algorithm to solve the challenges of balancing jobs on available heterogeneous VMs. 

However, these algorithms have problems, such as being trapped in a locally optimal, having a high computational cost, a slow convergence rate, and being unsuitable for certain decision variables [20,21,22]. Compared to GA and ACO, PSO converges faster and provides a better solution because of its capacity to explore optimal solutions [23,24]. Therefore, variants of PSO are utilized for benchmarking the proposed technique.

Initially, the standard SOS was intended to solve persistent numerical optimization problems [25]. The SOS algorithm searches for a suitable species through symbiotic interactions. It simulates the symbiotic relationship between organisms that helps them to survive and propagate within the ecosystem. The SOS method takes into account a successive search space in order to perform a successful numerical optimization. As a result, this algorithm considers the population of organisms to find distinct areas from the stated search space to identify the global optimum solution. An initial population is established in the searching space by a group of randomly generated organisms. Each organism corresponds to a possible solution, which is most likely related to a fitness value representing the organisms’ level of adaption within their living environment. The biological interaction of organisms can be employed to determine variations in their life levels in each circumstance, increasing the adaptability degree and the likelihood of the ecosystem’s durability. Current numerical solutions would be determined in a virtual ecosystem using these organism’s changes or adaptations to improve fitness levels. Since its inception, the SOS algorithm has been modified multiple times to provide optimal and efficient solutions to various optimization problems. Some researchers have provided an enhanced version of SOS that includes changes to the phases and operators. These modified algorithms’ implementations have been found in the literature, given in references [21,26,27,28,29,30,31,32]. In the work of [16], a discrete variant of SOS (DSOS) is presented for tackling job scheduling problems in cloud computing environments. Utilizing the CloudSim tool kit, the proposed technique for transforming continuous solutions into discrete types was used to schedule independent tasks. Similarly, a variant of the SOS algorithm for modifying the mechanism of organism selection has been presented by [33]. The proposed technique chose three organisms from the ecosystem that did not have a preset symbiotic relationship to maximize the likelihood of obtaining an enhanced organism after a symbiotic interaction. 

SOS, unlike most metaheuristic algorithms, is parameter free, which is useful for its applications. However, compared to standard metaheuristic algorithms, SOS is a recent approach with a few modified variants of successful applications. SOS’s capacity to find a globally accepted solution to optimization problems makes it appealing for further exploration and development. Thus, the quest for global solutions to optimization problems would have a reasonable likelihood of success by further modifying the ecosystem’s mutual characteristics between the distinct species to address the imbalance problem among the heterogeneous cloud resources (VMs). Therefore, this study presents a G_SOS algorithm, a scheduling method based on a modified SOS for the best feasible mapping of different tasks to cloud resources to minimize task processing time, cost of resource usage, and optimal usage of cloud resources. The technique employs equity represented by the geometric mean of the randomly selected organisms to address the mutual characteristics of the organisms in the mutualism phase to improve the imbalance among the heterogeneous resources.

The following are the contribution of this work:

- The formulation of an optimal solution scheduling optimization technique method for minimizing makespan and degree of imbalance among VMs in the IaaS cloud.
- The design and implementation of the modified SOS algorithm tagged G_SOS for task scheduling in the IaaS cloud.
- The replacement of the traditional SOS algorithm relationship characteristics between two distinct organisms from an arithmetic mean to a geometric mean concept in order to enhance search diversity and global convergence.
- The evaluation of the technique’s performance indicators, which include makespan, cost, responsiveness, and the degree of imbalance among VMs.

Further discussions in this paper are arranged as follows: Section 2 introduces the related literature; Section 3 presents the problem formulation; Section 4 briefly introduces the Geometric Mean-Based Symbiotic Organisms Search (G_SOS) algorithm; the simulation and results analysis are presented in Section 5. This paper’s work is concluded in Section 6.

## 2. Related Works

### 2.1. Metaheuristic Techniques Used in Cloud Task Scheduling

Metaheuristics techniques are based on analogues of biological concepts. It has been demonstrated that metaheuristic-based strategies can achieve near-optimal solutions in a reasonable amount of time for some complex problems [2,34]. Metaheuristic approaches have been used to address resource scheduling difficulties, such as makespan and response time reduction. The methods have been shown to find an optimal mapping of tasks to resources, reduce computation costs, improve service quality, and increase computing resource utilization. Metaheuristic algorithms have demonstrated exceptional effectiveness in delivering near-optimal scheduling solutions for complex large-size problems and, as such, have piqued the interest of various researchers [8,35]. Nevertheless, metaheuristic algorithms continue to suffer from being trapped in local optima, premature convergence, delayed convergence, and imbalance between the search methods [20,21,22,36].

### 2.2. Symbiotic Organisms Search Technique (SOS)

The Symbiotic Organisms Search algorithm emulates the symbiotic relationship that organisms must uphold to survive and grow in the ecosystem. Cheng and Prayogo [24] applied the SOS (Symbiotic Organisms Search) algorithm to handle statistics, engineering design, and optimization problems. The results show that the algorithm performs exceptionally well in several complex numerical problems.

Since its conception, the SOS algorithm has been continually modified to offer optimal and efficient solutions to diverse optimization challenges [37]. It is worth noting that most of the improvements to the original SOS algorithm were achieved by reworking either the mutualism phase or the commensalism phase, or both. Except under exceptional circumstances is a fourth phase added to the original three.

In Abdullahi et al. [16], a discrete variant of the Symbiotic Organism Search (SOS) algorithm termed DSOS has been proposed. The proposed technique uses the Symbiotic Organisms Search (SOS) in the cloud to find the best schedules for tasks based on the available cloud resources. The results of the simulation process indicate that the algorithm outperforms PSO and can be used to solve large-scale scheduling issues.

Nama S. et al. [38] suggested an Improved Symbiotic Organisms Search (I-SOS) to solve various dynamic unconstrained global optimization problems. The technique employs a random weighted reflective parameter and a predation phase to improve its performance. The improved SOS experimental outcomes proved to have better efficiency compared to the PSO, DE, and SOS algorithms.

A Modified Symbiotic Organisms Search (MSOS) algorithm was proposed by S. Banerjee and S. Chattopadhyay [27,39]. The algorithm changes the composition of the organism and chooses a set of parameters for a newly created symbiotic organisms search (SOS) algorithm to improve the convergence rate and its accuracy. MSOS splits the ecosize into three inhabitants, and the combined inhabitant is executed based on predefined probabilities. A new relationship is introduced into the phases to enhance the capacity to locate a stable and high-quality solution in a shorter period. The MSOS algorithm is endowed with a chaotic element created by the logistic map to find the most promising zones.

Tejani et al. [40] proposed an algorithm for optimizing structural design using adaptive symbiotic organisms’ search (SOS). The proposed technique modifies the benefit factors of SOS based on the fitness value of the current organism and the best organism’s fitness value. Furthermore, the benefit factor improved the exploration capability, especially when the two distinct organisms $X_{i}$ or $X_{j}$ (I ≠ *j*) are far from the best ($X_{best}$) in the search space. In the same vein, when the two distinct interaction organisms are closer to the best organism, it strengthens its exploitation capability.

A revised symbiotic organisms search algorithm for the problem of unmanned combat aerial vehicle (UCAV) route planning was presented by F. Miao et al. [41]. Under a multi-constrained global optimization problem, the proposed technique modified the standard SOS based on the simplex method (SMSOS). The method improves population diversity, thereby overhauling intensification and diversification to avoid premature convergence and local optima entrapment. An improved discrete symbiotic organism search technique for efficient cloud task scheduling has been presented by [42]. The proposed approach (eDSOS) enhances the diversification of the local search space by replacing the best value with any candidate in the ecosystem at the mutualism level of the DSOS algorithm, allowing it to converge much faster when the search space is more extensive.

However, most of these modified SOS algorithms still face the perennial problems of becoming stuck at the optimal local region of the search space, slow convergence rate, and imbalance among the cloud resources resulting in poor performance.

### 2.3. The Standard Symbiotic Organisms Search (SOS) Algorithm Procedure

The Standard Symbiotic Organisms Search (SOS) Algorithm that imitates the symbiosis relationship among distinct species in the ecosystem was presented by [25]. In this technique, the generation of new solutions is guided by mimicking the biological interaction between two species in the ecosystem. The technique has three phases of Mutualism, Commensalism, and Parasitism, through which each species interacts with the other species at random until the termination criteria are reached. The positions of the organisms at the iteration stage are updated by imitating the three phases of symbiotic relationships (mutualism, commensalism, and parasitism). The basic pseudocode of classical/traditional SOS is depicted in Algorithm 1.

**Definition** **1.** *Given a function f: Ա → ℛ find $X^{\prime }$ ∈ Ա: ∀ X ∈ Ա $f\left(X^{\prime }\right)$ ≤ or ≥ f(X). ≤ (≥) minimization (maximization), where f is an objective function to be optimized and Ա represents the search space with the elements of Ա being the feasible solutions. x is a vector of optimization variables with the values X = {$x_{1},\;x_{2},\;x_{3},\;\ldots ,\;x_{n}$}. An optimal solution is a feasible solution $X^{\prime }$ that optimizes f.*

The procedure of SOS is described in more detail as follows:

**1st** **Step:** Ecosystem creation and initiation

The ecosystem’s initial population is generated and other control variables, such as ecosystem size and the maximum number of iterations. Real numbers are used to indicate the positions of the organisms in the solution space.

**2nd** **Step:** Choosing the organism with the best-fitting objective function, denoted as

$${ℛ}^{best}$$

**3rd** **Step:** Mutualism phase

In the Mutualism Phase of SOS, for each organism ${ℛ}_{i}$, an organism ${ℛ}_{j}$, with *j* ≠ *i*, is randomly chosen from the population to interact with the organism ${ℛ}_{i}$. Both organisms benefit from a mutualistic symbiotic relationship. New candidate solutions for both organisms ${ℛ}_{i}$ and ${ℛ}_{j}$ are calculated using (1) and (2), respectively.

(1) $${ℛ}_{i}^{\prime }={ℛ}_{i}+\gamma^{\prime }*\left[{ℛ}^{best}-(M_{v}*BF^{-1}\right)]$$

(2) $${ℛ}_{j}^{\prime }={ℛ}_{j}+\gamma^{\prime \prime }*\left[{ℛ}^{best}-(M_{v}*BF^{-2}\right)]$$

where *γ*′ and *γ*″ are uniformly generated random numbers between 0 and 1. The joint relational vector between organisms ${ℛ}_{i}$ and ${ℛ}_{j}$ denoted by $M_{v}$ and benefit factors $BF^{-1}$ and $BF^{-2}$ are evaluated using (3)–(5), respectively.

(3) $$M_{v}=\frac{{ℛ}_{i}+{ℛ}_{j}}{2}$$

(4) $$BF^{-1}=1+round\left(\gamma^{\prime }\right)$$

(5) $$BF^{-2}=1+round\left(\gamma^{\prime \prime }\right)$$

The new species ${ℛ}_{i}^{\prime }$ *and* ${ℛ}_{j}^{\prime }$ are generated by modelling their structures from $M_{v}$ and BF corresponding to the best organism ${ℛ}^{best}$ of the current population. While $M_{v}$ signifies the association of mutual relationships between the distinct organisms, BF, on the other hand, signifies the benefit level achieved by each species from their interaction. *γ*′ and *γ*″ are functions of randomly generated numbers between 0 and 1 that follow a uniform distribution. The fitness values of these new species $f({ℛ}_{i}^{\prime })$ *an* $f({ℛ}_{j}^{\prime }$) are evaluated and compared to each predecessor to select the fittest in the population. For instance, the fitness functions $f({ℛ}_{i}^{\prime })$ *and* $f({ℛ}_{j}^{\prime }$) are evaluated, ${ℛ}_{i}$ is updated to ${ℛ}_{i}^{\prime }$ using (1) if and only if $f({ℛ}_{i}^{\prime })$ is greater than $f\left({ℛ}_{i}\right)$ and ${ℛ}_{j}$ is equally updated to ${ℛ}_{j}^{\prime }$ if $f({ℛ}_{j}^{\prime }$) is greater than $f\left({ℛ}_{j}\right)$ as shown in (2). Note that the worst fitness values are replaced.

**4th** **Step:** Commensalism phase

In the ith iteration, an organism ${ℛ}_{j}$ is randomly selected from the ecosystem to interact with ${ℛ}_{i}$ where i≠j. While ${ℛ}_{j}$ is neutrally affected in their relationship, ${ℛ}_{i}$ benefits. The commensalism interaction is modelled according to (6).

(6) $${ℛ}_{i}^{\prime }={ℛ}_{i}+\gamma^{\prime }*({ℛ}^{best})$$

where *γ*′ is a uniformly generated random number between −1 and 1. Therefore, the fitness function $f\left({ℛ}_{i}^{\prime }\right)$ is evaluated and ${ℛ}_{i}$ is updated to ${ℛ}_{i}^{\prime }$ using (6) if and only if $f\left({ℛ}_{i}^{\prime }\;\right)$ is greater than $f\left({ℛ}_{i}\right).$

**5th** **Step:** Parasitism phase

In the ith iteration, a parasite vector ${ℛ}^{P}$ is created by modifying ${ℛ}_{i}$ with a randomly generated number in the range of the decision variables under consideration, and an organism ${ℛ}_{j}$ with i≠j is chosen at random from the population to act as host to the parasite ${ℛ}^{P}$. If the fitness value $f\left({ℛ}^{p}\right)$ is greater than the fitness value $f\left({ℛ}_{i}\right)$, then ${ℛ}^{P}$ will replace ${ℛ}_{i}$; otherwise, ${ℛ}^{P}$ will be discarded.

Steps 2–5 are performed until the stopping criterion is met.

**6th** **Step:** Termination/Stopping criterion

**Algorithm 1:** Traditional Symbiotic Organisms Search AlgorithmCreate and Initialize the Population of Organisms in the Ecosystem ℛ$=\left\{{ℛ}_{1},\;{ℛ}_{2},\;{ℛ}_{3},\;\ldots ,\;{ℛ}_{N}\right\}$ **Set the stopping the criteria** $\;iteration_{number}\;\leftarrow 0$ ${ℛ}^{best}\;\leftarrow \;$0 **Do** $\;iteration_{number}\leftarrow iteration_{number}+1$ i←0 **Do** i←i+1 j=1 to N $\;if\;f\left({ℛ}_{j}\right)>f\left({ℛ}^{best}\right)$ $\;{ℛ}^{best}\leftarrow {ℛ}_{j}$ **Mutualism phase** **Commensalism phase** **Parasitism phase** **While**
i<=N **While** the stopping criteria is false.

## 3. Problem Formulation

In a cloud computing environment, users’ various tasks are dynamically assigned to the desired virtual machine (VM) or cloud resources. Tasks to be scheduled are sent to the cloud broker by cloud users who query the cloud information service (CIS), a register containing the datacenter specifications regarding the available services that can be deployed to perform the job. It is assumed that tasks $T=\left\{T^{1},T^{2},T^{3},\;.\;.\;.,T^{n}\right\}$ are received by the cloud broker (CB) in a particular time interval and the processing elements (Virtual Machines), which are heterogeneous in nature because of their varied processing power, thus increasing the makespan of specific VMs while reducing the makespan of others. In the same sense, the cost of processing tasks in each VM varies. Suppose there are m virtual machines $\left\{VM^{1},VM^{2},VM^{3},\;.\;.\;.,VM^{m}\right\}$ at the datacenter when the cloud broker receives the tasks. The primary purpose of this work is to identify the best schedule for executing a group of tasks in VMs in the shortest time possible, while also improving the percentage of resource utilization, i.e., to schedule tasks on available VMs to achieve a minimal makespan with high resource utilization. Hence, the proposed method leverages the Expected Time to Compute (ETC) of the jobs to be scheduled on each VM to determine scheduling decisions. Based on the number of instructions per second (MIPS) of a VM and the task’s duration or length, ETC values are calculated, and the values are usually represented in matrix form as shown in Table 1. *ETC* matrix is an (n × m) that keeps track of how long each task takes, i.e., $T^{i}$ on each $VM^{j}$. It is measured as the ratio of Task length to the virtual machine capacity. 

Let $ETC^{i,j}\;i=1,2,3,\ldots ,n,\;j=1,2,3,\ldots ,m$ be the processing time of executing task $T^{i}$ on each virtual machine $VM^{j}$. It is the ratio of the task length $T^{i}$ measured in MI (millions of instructions) to the speed of the virtual machine $VM^{j}$ measured in MIPS (millions of instructions executed per second).

It is calculated as:

(7) $$ETC^{i,j}=Lenth\;of\;TaskT^{i}/capacity\;of\;VM^{j}=MI^{i}/MIPS^{j}$$

Assume the task $T^{i}$ start time is $ST\left(T^{i},VM^{j}\right)$ on the VM $VM^{j}$. The available time of $VM^{j}$ is denoted by push($VM^{j}$). Equations (8) and (9) define the starting and finishing time of a task $T^{i}$ on virtual machine $VM^{j}$.

(8) $$\mathrm{Therefore},\;ST\left(T^{i},VM^{j}\right)=\max\left\{push\left(VM^{j}\right)\right\}$$

Similarly, let the time of a task $T^{i}$ be $FT\left(T^{i},VM^{j}\right)$ on the VM $VM^{j}\;\mathrm{finish}$.

(9) $$FT\left(T^{i},VM^{j}\right)=ST\left(T^{i},VM^{j}\right)+ETC^{i,j}$$

Equation (10), which measures the strength of the organism’s degree of adaptation to the ecosystem, is used to calculate the fitness value of each organism.

(10) $$\;Objective\;Function=max\left\{\overset{m}{\underset{j=1}{\sum }}\frac{f\left(VM^{j}\right)}{m}\right\}$$

(11) $$f\left(VM^{j}\right)=\frac{Ɀ}{makespan}$$

(12) $$Ɀ=\overset{m}{\underset{j=1}{\sum }}\frac{{𝓇}^{j}}{m}\;$$

(13) $${𝓇}^{j}=\frac{Task\;T^{i}}{makespan}$$

(14) $$Makespan=\max\{ETC^{i,j}\;i\in T,\;i=1,2,3,\ldots ,n\;and\;j\;\in VM,\;j=1,2,3,\ldots ,m\}$$

While Equation (11) represents the virtual machine $VM^{j\prime }s$ fitness value, Equation (12) shows the average usage of virtual machines employed for task execution denoted by Ɀ. The utilization of virtual machine $VM^{j}$ is defined by ${𝓇}^{j}$, as indicated in Equation (13). Equation (14) defines the maximum time virtual machines work in parallel to accomplish the task.

The Degree of Imbalance (*DOI*) presented in Equation (15) is the load distributed among the total number of VMs according to their computing capabilities [43,44]. *DOI* is calculated using the following equation. Here, $T^{max},T^{min}$ and $T^{average}$ represent the maximum, minimum, and average execution times among all the VMs. The execution time of a VM is the total time that the VM is busy.

(15) $$DOI=\frac{T^{max}-T^{min}}{T^{average}}$$

Let $\left\{Pc^{1},\;Pc^{2},Pc^{3},\;.\;.\;.,Pc^{m}\right\}$ represent the unit cost of virtual machine $VM^{j}$ per time quantum [45,46].

The unit cost of executing a task $T^{i}$ on a $VM^{j}$ in this study is considered per second basis. 

The total cost of processing tasks $T^{i}$ through $T^{n}$ on the available virtual machines $VM^{j}$ through $VM^{m}$ is presented in Equation (16) below:

(16) $$TCost=\overset{n,m}{\underset{i=1.j=1}{\sum }}\{C^{i,j}*Pc^{j}\}$$

In this situation, the costs of data transmission and retrieval are ignored because the tasks are independent [47,48].

The cost matrix is a 1 × *n* matrix that contains each virtual machine’s cost. The values of the expected time of completion (ETC) matrix and the cost matrix are normalized by dividing them by their respective maximum values [49,50].

Therefore, since the virtual machines are heterogeneous in nature, the related cost of using each VM varies. The response time of a processor/machine is the time interval between the task **submission** time and the starting time of task execution [51]. It is the summation of the difference between submission time and waiting/delay time.

Response Time=(Start time−Submission time)

Therefore, the total response time denoted by $TResp^{Time\;}$ is presented in Equation (17) and is calculated thus:

(17) $$\;TResp^{Time}=\overset{n}{\underset{i=1}{\sum }}(Str^{Time^{T^{i}}}-Sub^{TimeT^{i}})\;$$

The goal of the scheduling problem, according to the essential characteristics of task scheduling in a cloud computing context, is to map every task $T^{i}$ vigorously *i* = 1, 2, 3, …, *n* to a suitable cloud virtual machine $VM^{j}$ *j* = 1, 2, 3, …, *m* to reduce overall execution time, cost, and response time, maximize resource utilization, and minimize the Degree of Imbalance (*DOI*) among the scheduling VMs absolutely.

### Correlation Coefficient

The correlation coefficient between two variables or metrics (makespan and cost) is calculated using the correlation coefficient function denoted by r in Equation (18). The correlation coefficient between variables *x* and *y* is given as

(18) $$r=Cor\left\{x,y\right\}=\frac{\sum \left(x_{i}-\bar{x}\right)\left(y_{i}-\bar{y}\right)}{\sqrt{\sum {\left(x_{i}-\bar{x}\right)}^{2}\sum {\left(y_{i}-\bar{y}\right)}^{2}}}$$

where r = correlation coefficient, the numerator of the expression represents the covariance, and the denominator represents the standard deviation., i.e., r is the ratio of covariance to standard deviation.

$x_{i}=$ values of x—variable in a sample

$\bar{x}=$ mean of the values of the x—variable

$y_{i}=$ values of y—variable in a sample

$\bar{y}=$ mean of the values of the y—variable

The correlation coefficient between makespan and cost using an instance of heterogeneous virtual machines with a specific workload or task shows how strong or weak is the relationship between the metrics (makespan and cost). The value of r is measured between (−1, 1). If r = 0, it indicates no relationship between the two variables or metrics, meaning any increase or decrease in makespan will not have any effect on the cost. However, if r = +1 or close to 1, it indicates a strong positive relationship, meaning an increase or decrease in makespan will translate into an increase or decrease in cost. Similarly, if r = −1, it indicates a strong negative relationship, meaning an increase in makespan will lead to a decrease in cost, and a decrease in makespan will lead to an increase in cost. The two principal partners in the cloud, the cloud users or consumers and cloud providers, all have their objectives which are conflicting in nature. The cloud users want their jobs performed on time with minimum cost, while the cloud provider wants a judicious utilization of its resource to maximize profit or break even. For a job to be completed on time to attract more cost, meaning the smaller the processing time or makespan, the higher the cost incurred by the cloud service provider to provide such a high-powered system and the higher the cost of processing. This shows a robust negative relationship between makespan and cost.

## 4. Modified Symbiotic Organisms Search Algorithm (G_SOS)

The improvement of the SOS algorithm was informed by the relationship characteristics of the mutual vectors between two distinct organisms ${ℛ}_{i}$ and ${ℛ}_{j}$ in the Mutualism phase, calculated using the arithmetic mean, which signifies equality between the two species. However, in a heterogeneous cloud environment, the cloud resources likened to two distinct species can never have the same structure in terms of the processor’s speed, memory size, and storage capacity. Therefore, the proposed technique presents the mutual vector, which signifies relationship characteristics between organisms ${ℛ}_{i}$ and ${ℛ}_{j}$ that can be computed using the geometric mean concept to signify equity between the two species or resources.

A mutual vector ($M_{v}$) denotes a shared trait shared by two different species to improve their chances of survival or sustainability.

Where $M_{v}=\frac{{ℛ}_{i}+{ℛ}_{j}}{2}$, i.e., the arithmetic mean. 

Therefore, the geometric mean (Gm) for n number of organisms denoted by gm is shown in Equation (19).

(19) $$gm=\sqrt[{n}]{\left\{{ℛ}_{1}*{ℛ}_{2}*{ℛ}_{3}*,\;.\;.\;.,{ℛ}_{n}\right\}}$$

The geometric mean for two distinct species, ${ℛ}_{i}$ *and* ${ℛ}_{j},$ is calculated as shown in Equation (20).

(20) $$\;gm={\left\{{ℛ}_{i}*{ℛ}_{j}\right\}}^{\frac{1}{2}}$$

Therefore, Equation (21) shows the mutual vector between the two distinct species, calculated as the square root of the absolute value of the product of ${ℛ}_{i}$ and ${ℛ}_{j}$.

(21) $$\;{ℛ}^{Mv}=\sqrt[{2}]{\left|\left\{{ℛ}_{i}*{ℛ}_{j}\right\}\right|}$$

Gm ensures equity in each species contribution to their survival. Each VM contributes its resource by its capability and strength to improve its mutualistic characteristics, increasing its survival advantage, increasing the exploration capability of the technique, thereby increasing the convergence speed of the search process to achieve a globally optimal solution.

### 4.1. Mutualism Phase

During this phase, two species ${ℛ}_{i}$ and ${ℛ}_{j}$, where (*i* ≠ *j*) are randomly chosen to interact with each other. The two separate species were mutually beneficial. Equations (22) and (23) represent the mathematical description of this technique, respectively:

(22) $$A={ℛ}_{i}+\gamma^{\prime }\times \left[{ℛ}^{best}-{ℛ}^{Mv}\times BF^{-1}\right]$$

(23) $$\;B={ℛ}_{j}+\gamma^{\prime \prime }\times \left[{ℛ}^{best}-{ℛ}^{Mv}\times BF^{-2}\right]$$

where *A* and *B* represent the two organisms selected randomly from the ecosystem. The mutual vector (${ℛ}^{Mv}$) and benefit factors ($BF{\;}^{-1}\;and\;BF^{-2}$) are derived from the respective mathematical formulation in Equation (10) and Equations (4) and (5) as described in the work of [36].

The new species ${ℛ}_{i\_new}^{\prime }\;and\;{ℛ}_{j\_new}^{\prime }$ are generated by molding their structure from ${ℛ}^{Mv}$ and BF (benefit factors), corresponding to the best organism $\left({ℛ}^{best}\right)$ of the current population as shown in Equations (24) and (25).

Modify the new organisms to reflect the discretization of the algorithm using

(24) $${ℛ}_{i\_new}^{\prime }=[A]\;mode\;m+1$$

(25) $${ℛ}_{j\_new}^{\prime }=[B]\;mode\;m+1$$

The fitness values of new species ${ℛ}_{i\_new}^{\prime }\;and\;{ℛ}_{j\_new}^{\prime }$ are evaluated and compared to each predecessor to select the fittest in the population.

If $f({ℛ}_{i\_new}^{\prime })<f({ℛ}_{i}^{})$

$${ℛ}_{i}\leftarrow {ℛ}_{i\_new}^{\prime }$$

Similarly, if $f\left({ℛ}_{j\_new}^{\prime }\right)<f\left({ℛ}_{j},\right)$

$${ℛ}_{j}\leftarrow {ℛ}_{j\_new}^{\prime }$$

Note that worst fitness values are replaced or rejected.

### 4.2. Commensalism Phase

Select a random organism ${ℛ}_{j}$, where ${ℛ}_{i}$ ≠ ${ℛ}_{j}$ and let C be the new status of the organism ${ℛ}_{i}$ with an acceptable range of [0.4, 0.9] as expressed in the work of [52,53] as against [−1, 1], which extends the search space with slow convergence speed to minimize computation time, increase the convergence speed and ensure better results as shown in Equation (26). Modify the new organisms to reflect the discretization of the algorithm using Equation (27):

(26) $$C={ℛ}_{i}+rand\left(0.4,0.9\right)\times \left[{ℛ}^{best}\;–\;{ℛ}_{j}\right]\;$$

(27) $$\mathrm{Additionally},\;{ℛ}_{i\_new}^{\prime }=[C]\;mode\;m+1$$

If $f\left({ℛ}_{i\_new}^{\prime }\right)<f\left({ℛ}_{i}\right)$

$${ℛ}_{i}\leftarrow {ℛ}_{i\_new}^{\prime }$$

### 4.3. Parasitism Phase

Select a random organism ${ℛ}_{j}$, where ${ℛ}_{i}\neq {ℛ}_{j}$, let D be the new status of the parasite vector created from the organism ${ℛ}_{i}$ as shown in Equation (28). Modify the new parasite vector D to reflect the discretization of the algorithm using Equation (29).

(28) $$D=\mathrm{rand}\left(0,1\right)\times {ℛ}_{i}$$

(29) $${ℛ}^{p}=\lceil D\rceil \mathrm{mode}\;m+1$$

If $f\left({ℛ}^{p}\right)<f\left({ℛ}_{j}\right)$

$${ℛ}_{j}\leftarrow {ℛ}^{p}$$

**Figure 1:**
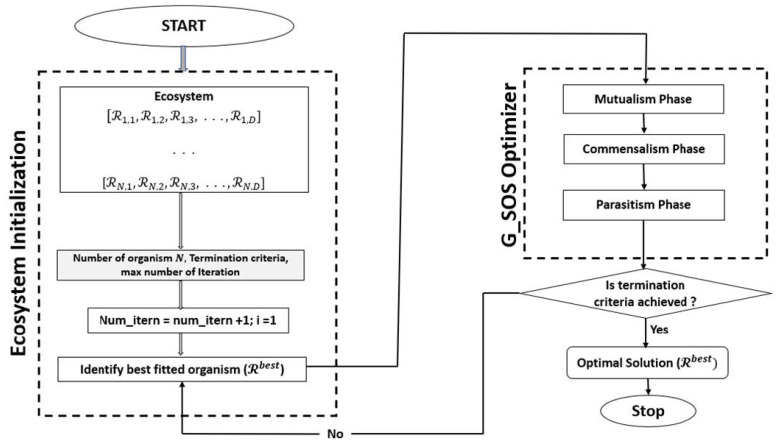
Flowchart of G_SOS Technique.

While the steps of the modified SOS algorithm (G_SOS) are described in Algorithm 2, Figure 1 shows the flowchart of the proposed G_SOS process/workflow.
**Algorithm 2:** Modified Symbiotic Organism Search algorithm (G_SOS) pseudocode**Input:** Size of population (ecosize), maximum number of iterations (Maxitern) **Output:**
${ℛ}^{best}$ is the optimal solution. **The Looping of G_SOS begins:** **While itern < maxitern**
For i = 1: Population (ecosize) For each species in the ecosystem ${ℛ}_{i}$, i = 1, 2, 3, …, ecosize, search for the organism with the best fitness value ${ℛ}^{best}$
**Mutualism Phase**Randomly select organisms ${ℛ}_{i}$ and ${ℛ}_{j}$(i≠j)Calculate the mutual vector (${ℛ}^{Mv}$) Equation (21) and the benefit factors ($BF{\;}^{-1}\;and\;BF^{-2}$) using Equations (4) and (5) as described in the work of [36]   Using Equations (24) and (25) to generate the new organisms ${ℛ}_{i\_new}^{\prime }\;and\;{ℛ}_{j\_new}^{\prime }$ and evaluate their fitness values.    If the new organisms’ fitness values are higher, then replace the predecessors
**Commensalism Phase** Select organism ${ℛ}_{j}$ randomly (i≠j)
  Using Equation (27) to generate a new organism ${ℛ}_{i\_new}^{\prime }$ and evaluate its fitness value   If the new organisms’ fitness values are higher, then replace the predecessor.
**Parasitism Phase** Select organism ${ℛ}_{j}$ randomly (i≠j)
 Generate parasite vector ${ℛ}^{p}$ by modifying ${ℛ}_{i}\;$ in Equation (29) Evaluate the fitness value   If the parasite vector $({ℛ}^{p}$)s’ fitness value is higher, then
$\mathrm{replace}\;{ℛ}_{j}$ with ${ℛ}^{p}$
**End for**  Update the best organism ${ℛ}^{best}$ of the current population (ecosize)**End while**

## 5. Simulation and Results

**Figure 2:**
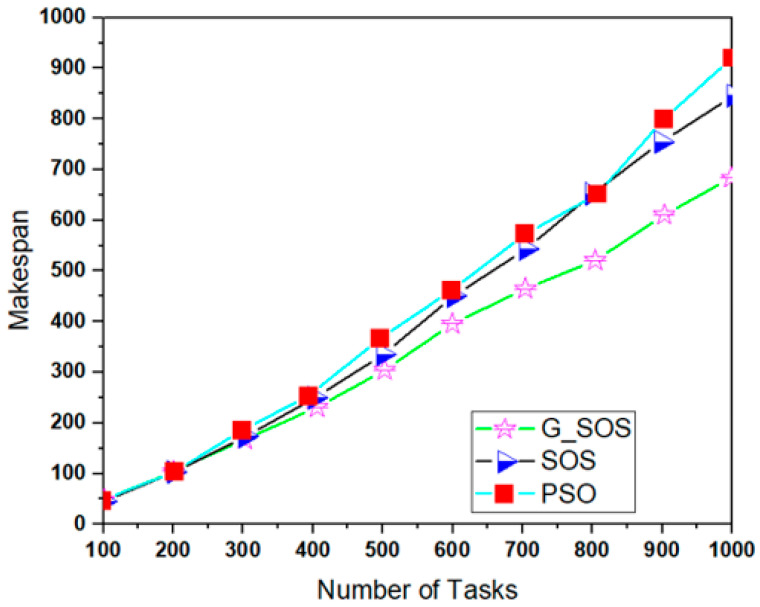
Makespan comparison between G_SOS, SOS and PSO (uniform distribution).

**Figure 3:**
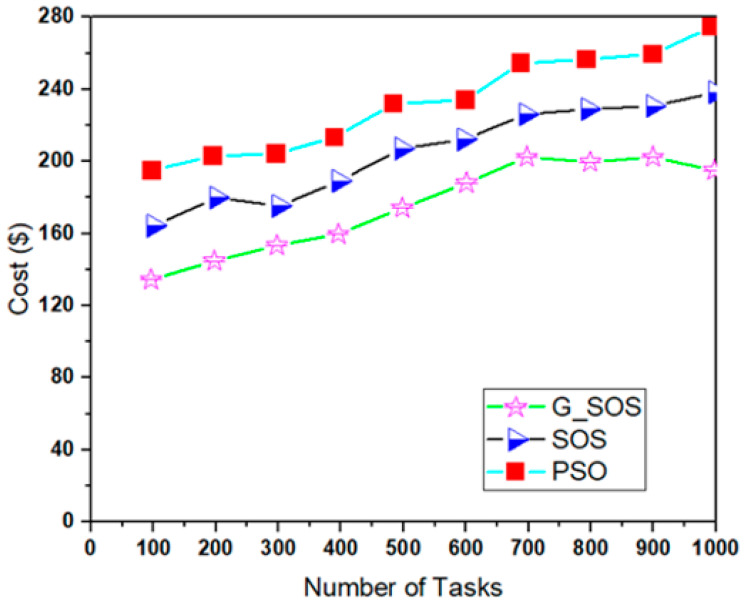
Average cost corresponding to the number of tasks.

**Figure 4:**
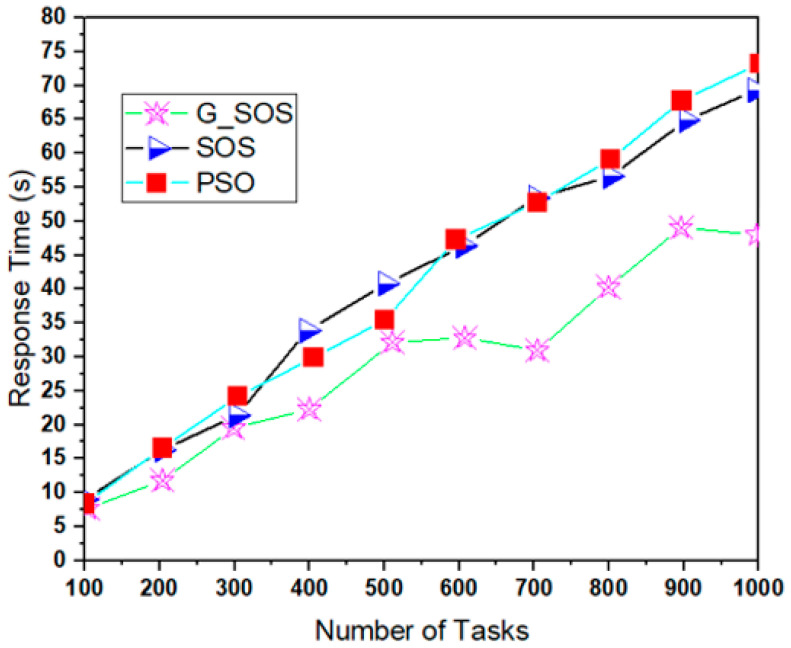
Response comparison between G_SOS, SOS and PSO (uniform distribution).

**Figure 5:**
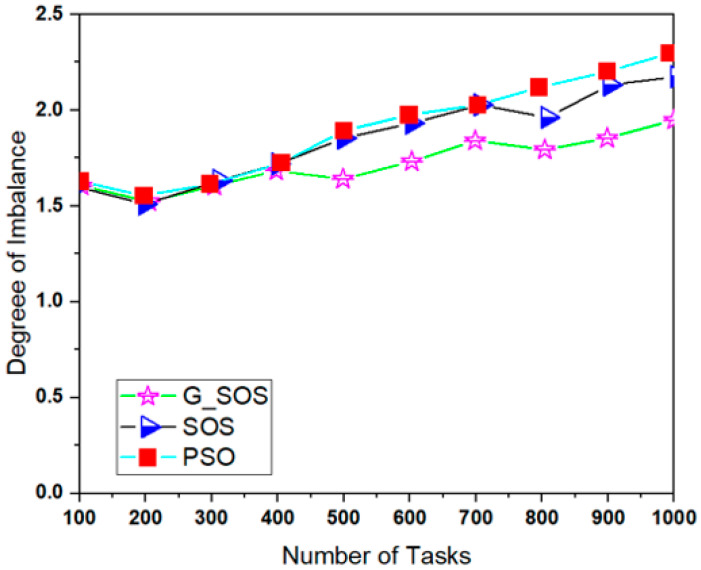
Degree of Imbalance comparison between G_SOS, SOS and PSO.

**Figure 6:**
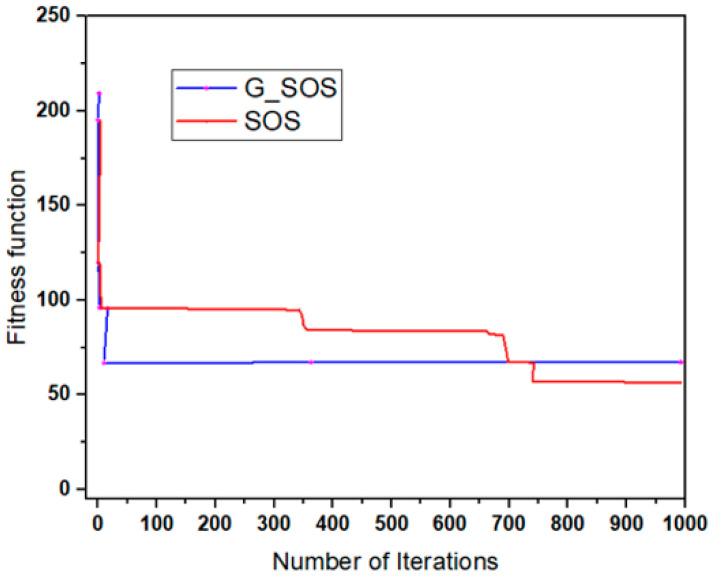
Convergence graph with sample tasks.

Simulations were run using the cloudsim-3.0.3 toolkit simulator to test the performance of the proposed technique [54,55,56,57,58]. The cloud environment was characterized by the heterogeneity of tasks and virtual machines. A single data center with two hosts was established. Each host had 1 TB of storage, 20 GB of RAM 10 Gbps of bandwidth, and a time-shared virtual machine scheduling technique. Twenty (20) virtual machines (VMs) were created, ten (10) each per host, each with a 10 Gigabyte image size, 0.5 Gigabyte memory, 1 Gigabyte per second bandwidth, and one processing element. The VMs ranged in processing power from 100 to 5000 MIPS, with prices ranging from USD 0.05 to 0.25. All the VMs were run on a time-shared cloudlet scheduler with Xen VMM. In 100, 200, 300, 400, 500, 600, 700, 800, 900, and 1000 examples; a consistent distribution of task sizes was generated, resulting in an equal number of large, medium, and small assignments. We can better grasp the algorithms’ scalability and performance when dealing with large problem sizes by using more significant instances. For each algorithm, the simulation was repeated for 40 experimental runs. Table 2 lists the algorithm parameter settings for the simulation experiment, while the CloudSim experimental settings are listed in Table 3. Figure 2, Figure 3, Figure 4, Figure 5 and Figure 6 show simulation results for the scheduling of G_SOS, SOS, and PSO algorithms with a task count ranging from 100 to 1000 for 40 runs each. As illustrated in Figure 2, Figure 3, Figure 4 and Figure 5, the average makespan, cost, response time and degree of imbalance of the proposed technique outperformed the traditional SOS algorithm and hybrid PSO-SA algorithm. G_SOS achieves the shortest makespan, lowest cost, minimal response time, as well as a minimal degree of imbalance. The statistical analysis of the performance of G_SOS, SOS and PSO-SA under the same data instances is presented in Table 4, Table 5, Table 6, Table 7, Table 8, Table 9, Table 10 and Table 11, which indicate that, for data instances of 100 through 1000, the calculated correlation coefficient between makespan and cost shows a strong positive relationship. This is deduced from the simulation results, which indicate that, as the size of the workload/tasks increases, both metrics of performance also increase in numerical strength.

G_SOS produces higher-quality solutions than regular SOS and PSO-SA, especially when the problem size is enormous. Figure 5 depicts G_SOS having the lowest degree of imbalance among the heterogeneous virtual machines than the traditional SOS and PSO-SA. Similarly, G_SOS’s search direction tends to converge to a stable position after fewer iterations, as shown in Figure 6. The new technique can be used alone or combined with other metaheuristic algorithms to address various optimization problems in cloud computing and other disciplines.

The correlation coefficient between two variables or metrics (makespan and cost) is calculated using the correlation coefficient function denoted by r in Equation (10). The two principal partners in the cloud, the cloud users, or consumers, and cloud providers, all have their own objectives which are conflicting in nature. The cloud users want their jobs performed on time with minimum cost, while the cloud provider wants a judicious utilization of its resources to maximize profit or break even. For a job to be completed on time to attract more cost, meaning the smaller the processing time or makespan, the higher the cost incurred by the cloud service provider to provide such a high-powered system and the higher the cost of processing. This shows a robust negative relationship between makespan and cost. However, in this scenario, the correlation coefficient between makespan and cost is 0.940691, showing a strong positive relationship. That is, as the workload size increases either on the same or different virtual machines, both metrics (makespan and cost) also increase.

## 6. Conclusions and Future Works

This paper presents a modified symbiotic organism search optimization algorithm called G_SOS motivated by the symbiotic relationship in organisms. The proposed method uses a modified SOS algorithm that changes the relation characteristic of species from the arithmetic mean, which signifies equality, to the geometric mean, which signifies equity, to address the heterogeneous nature of the cloud resources. The proposed technique was tested using the CloudSim tools to schedule independent jobs. The makespan, cost, response time, and degree of imbalance were measured, and G_SOS was shown to be better than the benchmarks, SOS and PSO-SA. According to simulation results, the proposed technique, G_SOS, outperforms regular SOS and PSO-SA in terms of convergence speed, cost, response time degree of imbalance, and makespan. The percentage of improvement of the proposed G_SOS over SOS and PSO-SA in terms of makespan minimization is 0.61% to 20.08% and 1.92% to 25.68%. Similarly, the percentage of improvement of the proposed G_SOS in terms of cost minimization over SOS and PSO-SA is 10.46% to 19.59% and 19.74% to 31.08%.

The response time minimization of the proposed technique (G_SOS) was achieved with 8.74% to 42.09% and 8.13% to 41.34% of SOS and PSO-SA improvement, respectively. Additionally, the Degree of Imbalance of G_SOS has a significant rate of improvement over SOS and PSO-SA with 1.26% to 13.04% and 0.55% to 15.79%. The obtained results validate the proposed G_SOS approach’s efficiency. The application of the proposed technique G_SOS is not limited to task scheduling problems. In the future, it can be extended to other domains, such as energy-aware task scheduling, scalability-aware task scheduling, engineering construction design, etc.

## Figures and Tables

**Table 1:** Expected time of completion (ETC) matrix.

T VM	$T^{1}$	$T^{2}$	-	-	$T^{n}$
$VM^{1}$	$T^{1}/VM^{1}$	$T^{2}/VM^{1}$	-	-	$T^{n}/VM^{1}$
$VM^{2}$	$T^{1}/VM^{2}$	-	-	-	$T^{n}/VM^{2}$
-	-	-	-	-	-
-	-	-	-	-	-
-	-	-	-	-	-
$VM^{m}$	$T^{1}/VM^{m}$	$T^{2}/VM^{m}$	-	-	$T^{n}/VM^{m}$

**Table 2:** Parameter settings for SOS and PSO.

Algorithm	Parameter	Value
SOS	Ecosize	100
	Number of iterations	1000
PSO	Particle size	100
	Static Inertial weight	0.9
	Variable Inertia weight, ⱳ	0.9–0.4
	Coefficients C_1 and C_2	2
	Number of iterations	1000

**Table 3:** Parameter settings for CloudSim.

Cloud Entity	Parameter	Value
Datacenter	Number	1
Host	Number	2
	Processing speed	1,000,000 MIPS
	RAM	20 GB
	Storage	1 Terabyte (TB)
	Bandwidth	10 GB/s
	Operating system	Linux
	Architecture	x86
	VMM	Xen
VM	Number	20
	Bandwidth	1 GB/s
	Memory	0.5 GB
	Image size	10 GB
	Processing speed (MIPS)	100–5000
	Scheduler	Time-shared
Task	Number of tasks	100–1000

**Table 4:** Makepan comparison between SOS and G SOS for data instances generated from a uniformly distributed dataset.

Number of Tasks	SOS	G_SOS	Improvement Rate (%)
100	45.2864	44.8002	1.07
200	102.8854	102.2623	0.61
300	173.3352	167.5982	3.31
400	249.2924	230.9342	7.36
500	335.1172	304.1376	9.24
600	450.3151	395.4699	12.18
700	543.2537	464.5428	14.49
800	651.5674	520.7649	20.08
900	754.3735	610.7203	19.04
1000	845.7058	683.9238	19.13

**Table 5:** Makepan comparison between PSO-SA and G SOS for data instances generated from a uniformly distributed dataset.

Number of Tasks	PSO-SA	G_SOS	Improvement Rate (%)
100	46.6633	44.8002	3.99
200	104.2623	102.2623	1.92
300	185.9565	167.5982	9.87
400	253.4230	230.9342	8.87
500	366.0967	304.1376	16.92
600	461.7890	395.4699	14.36
700	572.8564	464.5428	18.91
800	651.5674	520.7649	20.08
900	799.3512	610.7203	23.60
1000	920.2861	683.9238	25.68

**Table 6:** Cost comparison between SOS and G SOS for data instances generated from a uniformly distributed dataset.

Number of Tasks	SOS	G_SOS	Improvement Rate (%)
100	164.3427	134.3689	18.24
200	179.9629	144.7120	19.59
300	175.3191	153.3664	12.52
400	189.1450	159.6989	15.57
500	207.0871	174.1581	15.90
600	212.2586	187.9840	11.44
700	226.0846	202.4432	10.46
800	229.0397	199.5936	12.86
900	230.7284	202.4432	12.26
1000	238.2218	194.9498	18.16

**Table 7:** Cost comparison between PSO-SA and G SOS for data instances generated from a uniformly distributed dataset.

Number of Tasks	PSO-SA	G_SOS	Improvement Rate (%)
100	194.9498	134.3689	31.08
200	203.0765	144.7120	28.74
300	204.2374	153.3664	24.91
400	213.4196	159.6989	25.17
500	231.8893	174.1581	24.90
600	234.2113	187.9840	19.74
700	254.4752	202.4432	20.45
800	256.6916	199.5936	22.24
900	259.6468	202.4432	22.03
1000	274.6337	194.9498	29.01

**Table 8:** Comparison of the Response Time of SOS and G_SOS for a uniformly distributed dataset.

Number of Tasks	SOS	G_SOS	Improvement Rate (%)
100	8.9932	7.6580	14.85
200	16.3056	11.7867	27.71
300	21.3792	19.5100	8.74
400	33.8679	22.3035	34.15
500	40.7900	32.1424	21.20
600	46.3771	32.8203	29.23
700	53.4431	30.9511	42.09
800	56.6269	40.2560	28.91
900	64.8842	49.0474	24.41
1000	69.2799	47.9793	30.75

**Table 9:** Comparison of the Response Time of PSO-SA and G_SOS for a uniformly distributed dataset.

Number of Tasks	PSO-SA	G_SOS	Improvement Rate (%)
100	8.3359	7.65801	8.13
200	16.5726	11.78667	28.88
300	24.1727	19.50995	19.29
400	29.8830	22.30347	25.36
500	35.4700	32.14243	9.38
600	47.3220	32.82027	30.64
700	52.7652	30.95108	41.34
800	59.1534	40.25598	31.95
900	67.8010	49.04737	27.66
1000	73.2648	47.97925	34.51

**Table 10:** Comparison of Degree of Imbalance between SOS and G_SOS for a uniformly distributed dataset.

Number of Tasks	SOS	G_SOS	Improvement Rate (%)
100	1.5908	1.5709	1.25
200	1.5496	1.5225	1.75
300	1.6315	1.6049	1.63
400	1.7180	1.6833	2.02
500	1.8529	1.6397	11.51
600	1.9312	1.7309	10.37
700	2.0265	1.8399	9.21
800	1.9612	1.7922	8.61
900	2.1308	1.8529	13.04
1000	2.1743	1.9482	10.40

**Table 11:** Comparison of the Degree of Imbalance between PSO-SA and G_SOS for a uniformly distributed dataset.

Number of Tasks	PSO-SA	G_SOS	Improvement Rate (%)
100	1.6267	1.5709	3.43
200	1.5532	1.5225	1.97
300	1.6138	1.6049	0.55
400	1.7228	1.6833	2.29
500	1.8917	1.6397	13.32
600	1.9741	1.7309	12.32
700	2.0265	1.8399	9.21
800	2.1178	1.7922	15.37
900	2.2002	1.8529	15.79
1000	2.2956	1.9482	15.13

## Data Availability

Uniform dataset: http://www.plosone.org/article/fetchSingleRepresentation.action? Uri=info: doi/10.1371/journal.pone. 0158229.s001 (accessed on 14 August 2021).

## Abbreviations

In this manuscript, the following abbreviations are used:

SOS	Symbiotic Organisms Search
G_SOS	Geometric-Based Symbiotic Organism Search
PSO	Particle Swarm Optimization
QoS	Quality of Service
SLA	Service Level Agreement
VM	Virtual Machine
MIPS	Million Instructions per Second
CB	Cloud Broker
CIS	Cloud Information Service
ETC	Expected Time to Compute
CPU	Central Processing Unit
UCAV	Unscrewed Combat Aerial Vehicle
eDSOS	Enhanced Discrete Symbiotic Organisms Search
DSOS	Discrete Symbiotic Organisms Search
SMSOS	Simplex Method Symbiotic Organisms Search
MSOS	Modified Symbiotic Organisms Search
I-SOS	Improved Symbiotic Organisms Search
